# Low-Field Optical Polarization in Type-II Quantum Dots via Nuclear-Driven Dark State Mixing

**DOI:** 10.1021/acs.nanolett.5c05163

**Published:** 2025-12-22

**Authors:** Gabriel M. Jacobsen, Vinicius A. de Oliveira, Baolai Liang, Morgan E. Ware, Gregory J. Salamo, Gilmar E. Marques, Yuriy I. Mazur, Victor Lopez-Richard, Marcio D. Teodoro

**Affiliations:** † Department of Physics, Federal University of São Carlos, 13565-905, São Carlos, São Paulo, Brazil; ‡ Institute for Nanoscience and Engineering, University of Arkansas, Fayetteville, Arkansas 72701, United States; § Department of Electrical and Computer Engineering, California NanoSystems Institute, University of California - Los Angeles, Los Angeles, California 90095, United States

**Keywords:** Type-II, Quantum dots (QDs), Optical polarization, State hybridization, Nuclear spins, Dark states

## Abstract

Semiconductor quantum dots (QDs) offer a rich landscape for spin control and quantum light emission. While most studies have focused on type-I band alignment, the potential of type-II systems remains underexplored. Here, we report low-field optical polarization in type-II In­(Ga)­As/GaAsSb QDs, enabled by hyperfine-induced mixing between bright and dark excitons via level anticrossing under magnetic fields as low as 0.17 T. The weak-field regime arises from the suppressed wave function overlap, yielding a reduced electron–hole exchange interaction. A theoretical model based on the spin Hamiltonian and the spin-split state populations accurately captures the observed mirror-symmetric luminescence helicity, reproducing the experimental polarization response. Additionally, polarization recovery measurements confirm the role of nuclear spin interactions in mediating the in-plane electron spin precession. Our work demonstrates an alternative route for light polarization control using weak magnetic fields and nonresonant linear excitation, establishing type-II QDs as promising platforms for compact sources of circularly polarized light.

## Graphical abstract

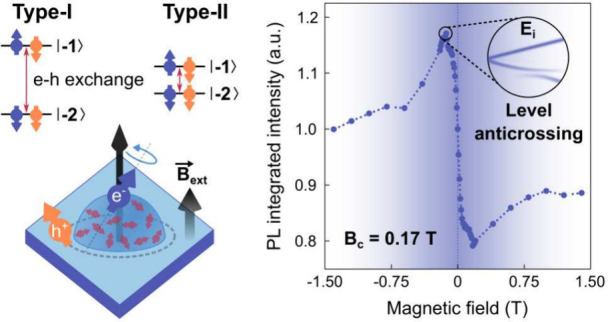

Semiconductor quantum dots (QDs) are pivotal material platforms for the development of spintronic and quantum technologies owing to their outstanding properties as quantum emitters—such as photon coherence, indistinguishability, and brightness^1–4^ as well as their long coherence times and high initialization fidelity, both essential for scalable spin qubits.^5–8^

Photon generation in QDs arises from the optical recombination of electron–heavy-hole pairs (or excitons) with opposite spin projections along the QD growth axis,^9,10^ forming the so-called bright states, typically represented in the total angular momentum basis as |*m*⟩_*z*_ = |±1⟩. In contrast, the optically inactive branch, known as dark states, consists of pairs with the same spin projections, corresponding to |±2⟩.^11,12^ The 4-fold degeneracy among these states is lifted by the electron–hole exchange interaction, prompting extensive investigations into the fine structure of excitons.^13–17^

Magneto-optical spectroscopy offers a powerful tool to investigate QD fine structure, as bright and dark states exhibit distinct Zeeman shifts depending on the sign and magnitude of the carriers’ *g*-factors. When magnetic fields are applied along the sample’s growth direction, for instance, weak perturbation potentials can induce in-plane magnetic components, enabling bright–dark mixing and the observation of level anticrossing (LAC) effects.^18–22^

The interplay between magnetic and electric fields and the exciton fine structure is also critical for developing circularly polarized photon sources. Traditionally, spin polarization in hybrid semiconductor structures has been achieved using ferromagnetic contacts.^23,24^ However, a recent proposal^25^ has introduced an alternative architecture for spin light-emitting diodes, exploiting the exciton fine structure and the hyperfine interaction to generate dynamic electron spin polarization.^26^ This scheme is particularly relevant in light of the growing interest in the effects imposed by the coupling between electron and nuclear spins in QDs.^27–31^

Quantum dot systems can also be engineered to exhibit type-I or type-II band alignments. While the former is well studied, type-II systems remain comparatively underexplored, despite recent reports of small and tunable fine structure splittings and promising implications for quantum gate applications.^32–35^ In type-I alignment, electrons and holes are confined within the nanostructure, resulting in large electron–hole wave function overlap and short optical recombination times. Conversely, type-II alignment occurs when only one carrier is confined, while the other is delocalized.^36,37^ Even though a smaller oscillator strength is expected, strong luminescence signals up to room temperature have been demonstrated.^38,39^ Furthermore, the longer carrier lifetimes and suppressed wave function overlap offer valuable characteristics for tailoring exciton fine structure and memory operations.^40–42^

Here, we demonstrate the generation of optical polarization in type-II InAs/GaAsSb QDs under magnetic fields as low as 0.17 T via level anticrossing and bright–dark mixing. The observation of the effect by photoluminescence measurements under weak fields originates from the reduced exchange splitting, a consequence of the small wave function overlap typical of the type-II alignment. A theoretical framework based on the spin Hamiltonian is developed, showing excellent agreement with the experimental data by accounting for the populations of the spin-split states. To identify the origin of the in-plane precession of electron spins, optical orientation was provided to probe the decoupling from nuclear spins via the polarization recovery curve, with the results aligning well with the LAC region.

While LAC effects have been previously reported in other systems,^19–21^ the typical outcome involves a unidirectional intensity enhancement of the bright state component, which consists of a single polarization channel of the ground state arising from carrier feeding through the dark reservoir. In contrast, our results reveal a clear and simultaneous mirror-symmetric response on the σ^+^ and σ^–^ circularly polarized components. We attribute this distinctive behavior to a delicate balance among the characteristic time scales of the system, formed by radiative and nonradiative recombination and spin lifetimes.

Our samples consist of InAs QDs capped by a GaAs_1–*x*_Sb_*x*_ strain-reducing layer, which have emerged as promising candidates for optoelectronic devices operating in telecom wavelengths. This potential arises from the surfactant effect of Sb, which preserves QD shape and height, and from the transition to a type-II band alignment for Sb compositions exceeding 14–17%.^32,43–47^

In order to demonstrate how the suppressed electron–hole wave function overlap in type-II QDs enables LAC effects at weak magnetic fields, we performed polarization-resolved magneto-photoluminescence (PL) measurements in two QD samples differing in Sb content within the GaAs_1–*x*_Sb_*x*_ layer: (i) one with *x* = 11%, labeled type-I QD, and (ii) one with *x* = 15%, labeled type-II QD. After, we focus the discussion on the type-II system.

Figure 1a illustrates the QD structure used for electronic structure simulations based on an 8-band **k**·**p** model within the Nextnano software.^48^ Diffusion of Ga and In atoms commonly leads to the formation of a ternary alloy in the QD region,^49^ which was included in the calculations (see Supporting Information). The 2D probability density colormaps in Figure 1b and Figure S1 reveal that, by increasing the [Sb] content in the capping layer in the 11–17% range, only weak variations are observed for the electron fundamental subband, remaining confined in the QD. Meanwhile, the heavy-hole probability density $\left(\Psi_{hh1}^{2}\right)$ exhibits significant modulation, mainly in the 11–15% interval when the probability maximum shifts to the lateral interfaces between the QD and GaAs_1–*x*_Sb_*x*_ layer, inducing the type-II alignment.

**Figure 1:**
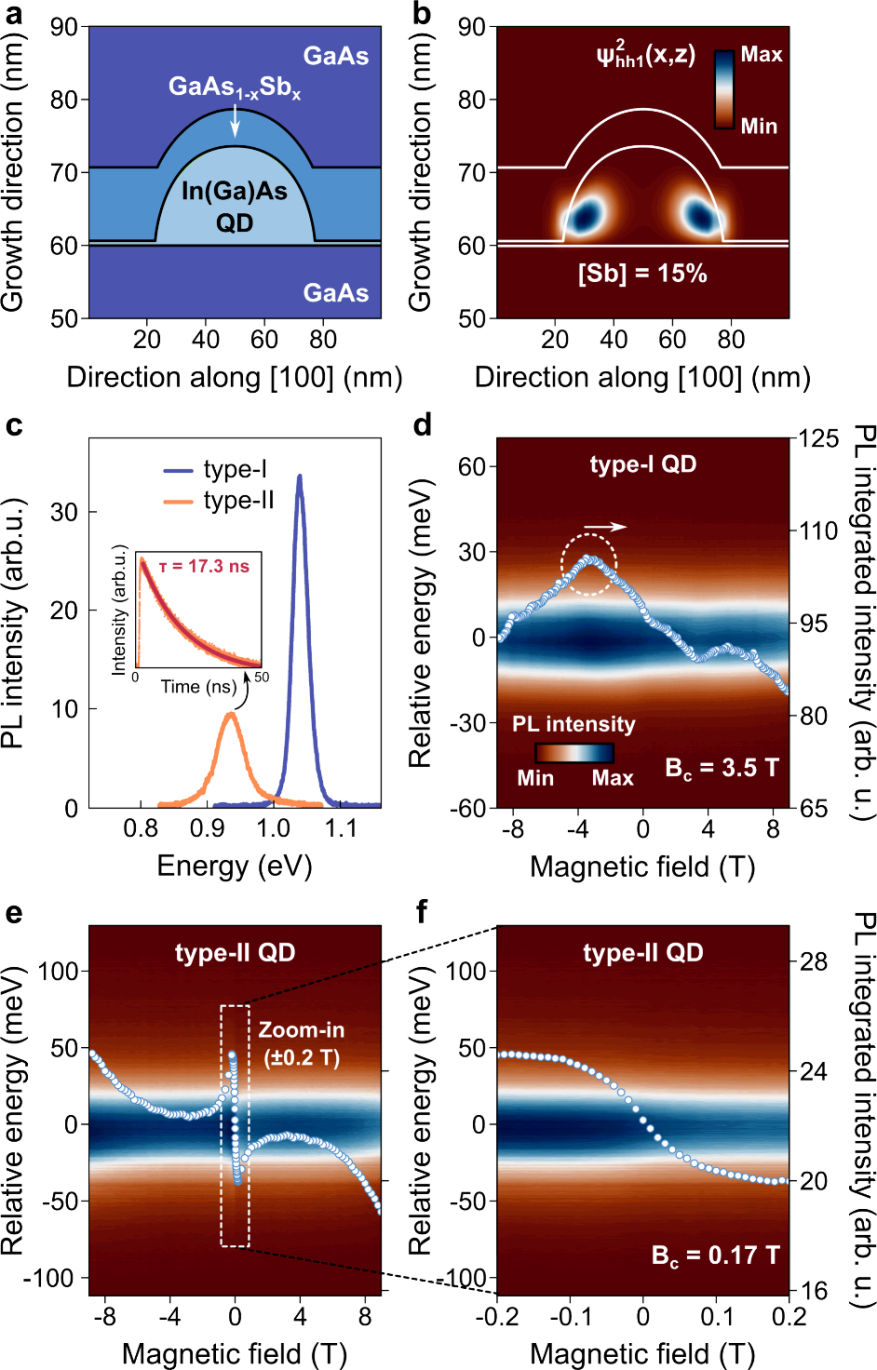
Photoluminescence from the type-I and type-II In­(Ga)­As/GaAs_1–*x*_Sb_*x*_ QDs. (a) QD structure obtained from the growth conditions and implemented in the electronic structure simulations. (b) 2D probability density colormap for the fundamental heavy-hole subband for *x* = 15%. Lines delineating the QD and GaAsSb regions are shown to guide the reader. (c) PL spectra for the type-I and type-II QDs at 3.7 K, excitation density of 250 W/cm^2^, and 0 T. The inset shows the luminescence decay for the type-II sample measured at its emission intensity maximum with the corresponding single-exponential decay fit. Circularly polarized 2D PL spectra ranging from −9 to 9 T at 3.7 K for (d) the type-I QD with 250 W/cm^2^ and (e) the type-II QD with 25 W/cm^2^. Open circles represent the integrated intensity (right *y*-axis) obtained from Gaussian fits to the data. A zoom-in of the low-magnetic-field region (±0.2 T) for the type-II QD is shown in (f). Integrated intensities in (e) and (f) are on the same scale.

The band alignment transition, combined with the preserved QD height during capping, leads to three experimental signatures: red-shifted luminescence, decreased PL intensity, and delayed recombination. These are evident in the PL spectra and time-resolved PL (TRPL) measurements in Figure 1c. By fitting the time decay of the type-II system with a single-exponential function, a recombination lifetime of 17.3 ns is found, whereas for type-I systems values of hundreds of picoseconds are generally obtained.^50^ A comprehensive optical characterization of both samples is reported in ref 51.

Magneto-PL colormaps for both QDs are shown in Figure 1d,e, with energy axes referenced relative to the PL peak at 0 T. The out-of-plane magnetic field is applied along the QD growth direction (*z*-axis), and the luminescence is analyzed in terms of the circularly polarized emissions, σ^+^ (positive fields) and σ^–^ (negative ones). We also added the integrated intensities obtained from Gaussian fits to the plots. For the type-I system, local PL intensity maxima and minima appear at −3.5 and 3.5 T, respectively. We define these positions by the parameter *B*_*c*_, as a reference to the level anticrossing point. For the type-II QDs, we observe two particular features: global maxima and minima at the extreme magnetic fields (±9 T), attributed to thermal redistribution,^52^ and local ones at *B*_*c*_ = ±0.17 T, as highlighted by the zoomed-in view in Figure 1f. We emphasize that these measurements were performed using linearly polarized, nonresonant excitation at 1.698 eV.

We attribute these remarkable intensity local maxima at strong and weak magnetic fields in the type-I and type-II QDs, respectively, to level anticrossing and mixing between bright and dark states. In this direction, we developed a theoretical framework based on the spin Hamiltonian, which accounts for the eigenenergies and projections onto the bright and dark state basis, and an exciton population dynamics model to determine the evolution of the circularly polarized light emission with the external magnetic field (see Supporting Information).

By introducing off-diagonal elements in the Hamiltonian through a perturbation potential, the effective magnetic field is moved from the *z*-axis, generating spin precession in the plane (*x*-*y*), which enables hybridization between bright and dark states. Consequently, anticrossing effects may emerge. Additionally, we reduced the framework to a three-level problem (two bright and one dark state), consistent with the observation of a single PL intensity peak/valley, prompting that only one anticrossing event occurs. Later, we justify it by a hyperfine-induced perturbation affecting only electron spins and by the preserved rotational symmetry in our QDs.

Figure 2a displays a schematic representation of the exciton fine structure and the magnetoluminescence in type-II QDs. The higher-energy and optically active bright states emit light with different helicity, namely, the σ^+^ (|+1⟩) and σ^–^ (|−1⟩) components. Conversely, the two lower-energy dark states have their recombination prohibited by the selection rules.^53^ The degeneracy among them is lifted by the combined action of exchange and Zeeman interactions. In our case, the energy splitting between bright states (δ_*b*_) is assumed negligible, since only small values, that do not influence LAC effects, are theoretically predicted for InAs/GaAsSb QDs.^32^ The energy dependence of the dark state in the unperturbed case is also added as a dashed orange line in Figure 2a.

**Figure 2:**
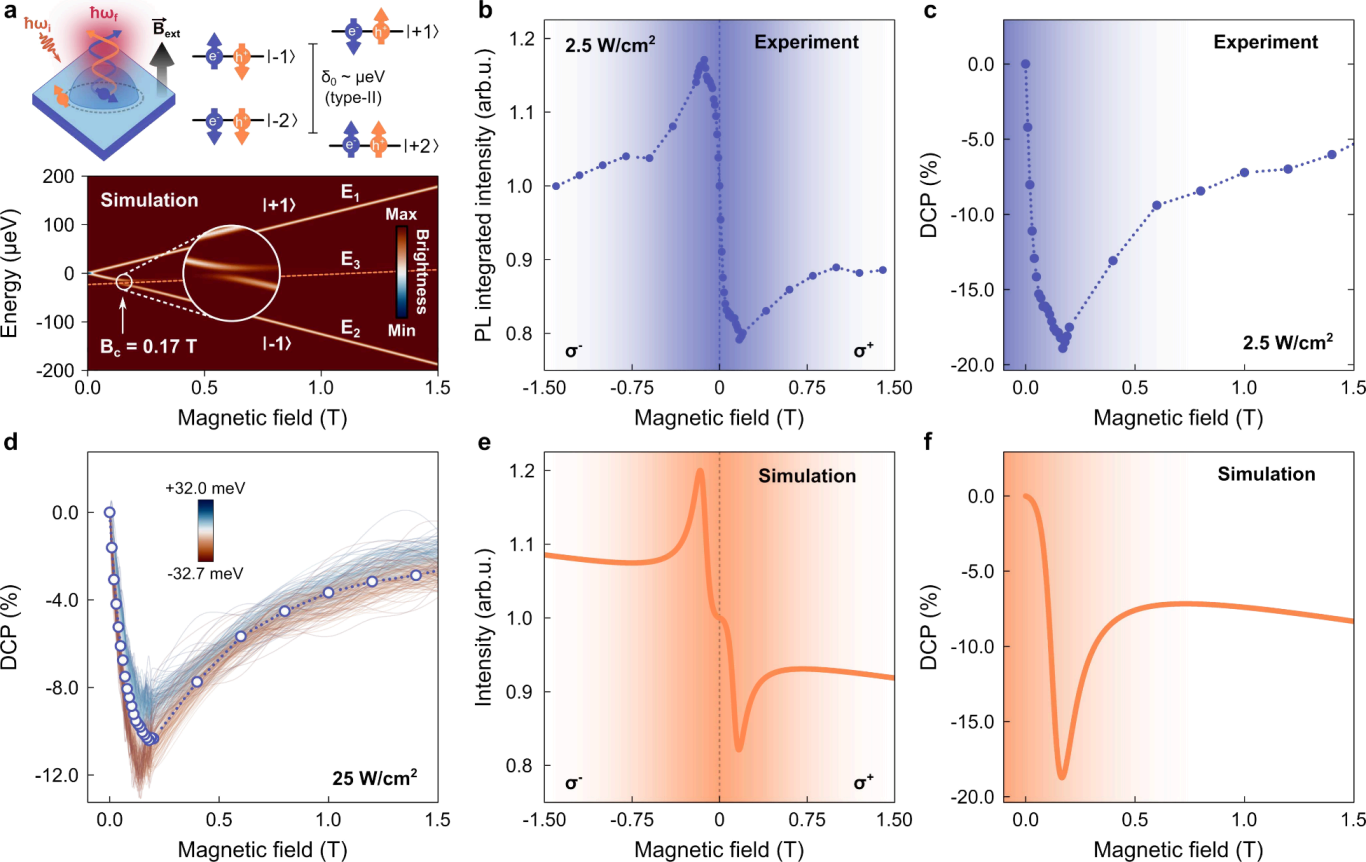
Level anticrossing (LAC) in the type-II QDs. (a) Schematic representation of the exciton fine structure, their luminescence, and the calculated 2D eigenenergy colormap weighted by the projections onto the bright states (|±1⟩). A zoomed-in view highlights the LAC region. The orange dashed lines represent the energy dependence of the unperturbed dark state. (b, c) Experimental PL integrated intensity and degree of circular polarization (DCP) as a function of the external magnetic field for 2.5 W/cm^2^. The intensities from the σ^+^ and σ^–^ circularly polarized components are resolved and correspond to experimental points at positive and negative magnetic fields, respectively. (d) Spectral dependence of the DCP from data points ranging between 900.08 and 964.84 meV with 0.22 meV steps for 25 W/cm^2^. The Gaussian-deconvoluted DCP points are also shown as a visual guide. (e, f) Simulated PL intensity and DCP as a function of the external magnetic field. Shaded backgrounds highlight the LAC region.

Solving the eigenvalue, *E*_*i*_, and eigenvector, *C*_*i*,*m*_, problem (eq 2 from the Supporting Information) allows the visualization of the LAC effect via a 2D eigenenergy map weighted by the bright state projections (*C*_*i*,±1_), as shown by our simulations in Figure 2a. Here, *i* = 1, 2, 3 represents the index of the three-level system, and *m* references the total-angular momentum basis set. The key element is that, while in traditional type-I In­(Ga)As QDs the magnitude of bright–dark splitting (δ_0_) lies in the hundreds of μeV,^54^ the exchange interaction suppression due to carrier spatial separation in type-II alignment drastically decreases it. Since the LAC field is given by $B_{c}=\delta_{0}/\left(\mu_{B}g_{e\left(h\right)}^{z}\right)$ ,^40^ reducing δ_0_ by the sole use of type-II QDs naturally shifts LAC to weak magnetic fields.

The experimental PL integrated intensity $\left(I_{\sigma^{\pm }}\right)$ and degree of circular polarization (DCP), defined as $DCP=\left(I_{\sigma^{+}}-I_{\sigma^{-}}\right)/\left(I_{\sigma^{+}}+I_{\sigma^{-}}\right)$ , for the type-II QDs are shown in Figure 2b,c for the ±1.5 T interval. The shaded areas highlight the LAC region. Remarkably, a very steep polarization curve is observed, achieving 18.9% optical polarization at 0.17 T, followed by a slow decay to 3.0% that persists until ∼4 T when thermal polarization begins to dominate (Figure S2). The identical polarization sign for both LAC- and thermally-induced polarizations confirms that the anticrossing involves the lower-energy bright state.

In the μ-PL measurements, we estimate that we collected the luminescence of 200 dots. To assess the possible influence from the QD ensemble, which may show a dispersion of *g*-factors and affect our conclusions,^55^ Figure 2d depicts the polarization spectral dependence by calculating the DCP for each point of the raw data within ∼±32 meV of the PL peak. The spacing between measured points is 0.25 meV, while a Zeeman shift of 0.24 meV is expected up to 1 T for an electron–heavy-hole pair *g*-factor of 4.1 (Figure S2). Hence, we certify that at least up to 1 T our spectral analysis involves the same dot distribution within the spectral resolution interval. As one may see, the LAC-induced polarization remains unchanged. The overall smaller DCP in comparison with Figure 2c is due to the higher excitation density.

Next, the eigenstate solutions are used as inputs to a population model (eqs 3–6 from the Supporting Information), and then, the intensity of the bright state emissions, described by $I_{\sigma^{\pm }}\propto \underset{i}{\sum }|C_{i,\pm 1}{|}^{2}f_{i}$ , with *f*_*i*_ representing the excitonic level population, and the DCP are calculated. The results are shown in Figure 2e,f and match well with the experimental data. Although the model includes an extensive list of parameters, each one of them has a very particular effect on the system.

The out-of-plane *g*-factors were determined from the experimental Zeeman splitting and the calculated electron *g*-factor based on ref 56. The radiative lifetime (τ_*r*_ = 17.3 ns) was extracted from the TRPL measurement. The nonradiative (τ_0_) and spin-flip (τ_*s*_) times were estimated by the interplay between the intensities of the simulated σ^+^ and σ^–^ components. In this case, the small contribution from thermal polarization in the low-field regime requires spin-flip times longer than the radiative recombination. At the same time, the mirror-symmetric LAC response, where a simultaneous intensity valley and peak occur for the emitted σ^+^ and σ^–^ light, demands a finite value for τ_*s*_ and an even longer one for the nonradiative part (τ_0_). Physically, this implies that the impacts of LAC in both bright states are only possible by considering spin relaxation into the population model and that the long-lived dark states do not nonradiatively recombine before electron and hole spins flip. In addition, dark state decay rates are expected to be much slower than for bright ones.^10,57^ Thus, we defined τ_*s*_ = 100 ns and τ_0_ = 1000 ns in our simulations.

The LAC field (*B*_*c*_) is determined by the exchange splitting, yielding δ_0_ = 23 μeV, which is significantly lower than the values expected in type-I QDs. The DCP peak amplitude is governed by the generation ratio between bright and dark states, *G*_*r*_/*G*_0_, estimated here as 0.4, which is appropriate for nonresonant excitation,^19^ and the relation between the recombination and spin dynamics of the system, requiring τ_*s*_ > τ_*r*_. The width of the LAC-induced polarization peak depends on the perturbation potential (*V*_*ii*′_), which must be proportional to the in-plane effective magnetic field component. We therefore consider $V_{23}\propto \mu_{B}B\sin\left(\theta \right)g_{e}^{x}$ , where θ is the angle between the QD growth direction (*z*) and the tilted electron spin precession axis and $g_{e}^{x}$ is the electron in-plane *g*-factor. Excellent agreement with the experimental data was obtained for θ = 30.0° and $g_{e}^{x}=2.0$ , consistent with the small *g*-factor anisotropy reported for similar systems.^58,59^ We also note that variations of only 5° in θ already result in substantial narrowing or broadening of the DCP peak for smaller or larger θ, respectively.

From the simulations, the perturbation potential (*V*_23_) in the spin Hamiltonian reaches 16.6 μeV,^21^ which corresponds to an effective fluctuating nuclear field *B*_*N*_ = 119 mT. This magnitude is fully consistent with the values typically reported for indium- or aluminum-based, momentum-direct QDs.^60–63^

We emphasize that the exceptional mirror-symmetric behavior between the σ^+^ and σ^–^ components, along with the considerable DCP peak, are the result of a very specific set of conditions among τ_*r*_, τ_0_, and τ_*s*_ within our type-II QDs, defining τ_0_ > τ_*s*_ > τ_*r*_. On one hand, if the spin lifetime is shorter than the optical recombination time, spin polarization would be dominated by the thermal contribution even at low magnetic fields, and the LAC-induced term would only contribute weakly. On the other hand, if τ_0_ < τ_*s*_, the mirror-symmetric response would be suppressed as only the bright component involved in the anticrossing would be affected by the mixing. For this reason, the influence of a single level anticrossing event in both polarization components has not been observed before, as either spin relaxation was not taken into account^19^ or equal τ_0_ and τ_*s*_ were considered.^21^ Furthermore, we highlight that reproducing the LAC-induced DCP peak is possible only by the combined characterization of the population dynamics of the system and the electronic structure of the hybridized states.

Finally, to identify the origin of the in-plane spin precession at low magnetic fields, we performed optical orientation measurements as a function of the magnetic field for the type-II QDs, with the results shown in Figure 3a,b. Briefly, optical polarization is induced via angular momentum transfer from circularly polarized photons to carrier spins through spin–orbit coupling.^64^ As the magnetic field applied along the growth direction increases, electron and hole spins are progressively decoupled from eventual in-plane components, leading to an enhancement of the DCP.

**Figure 3:**
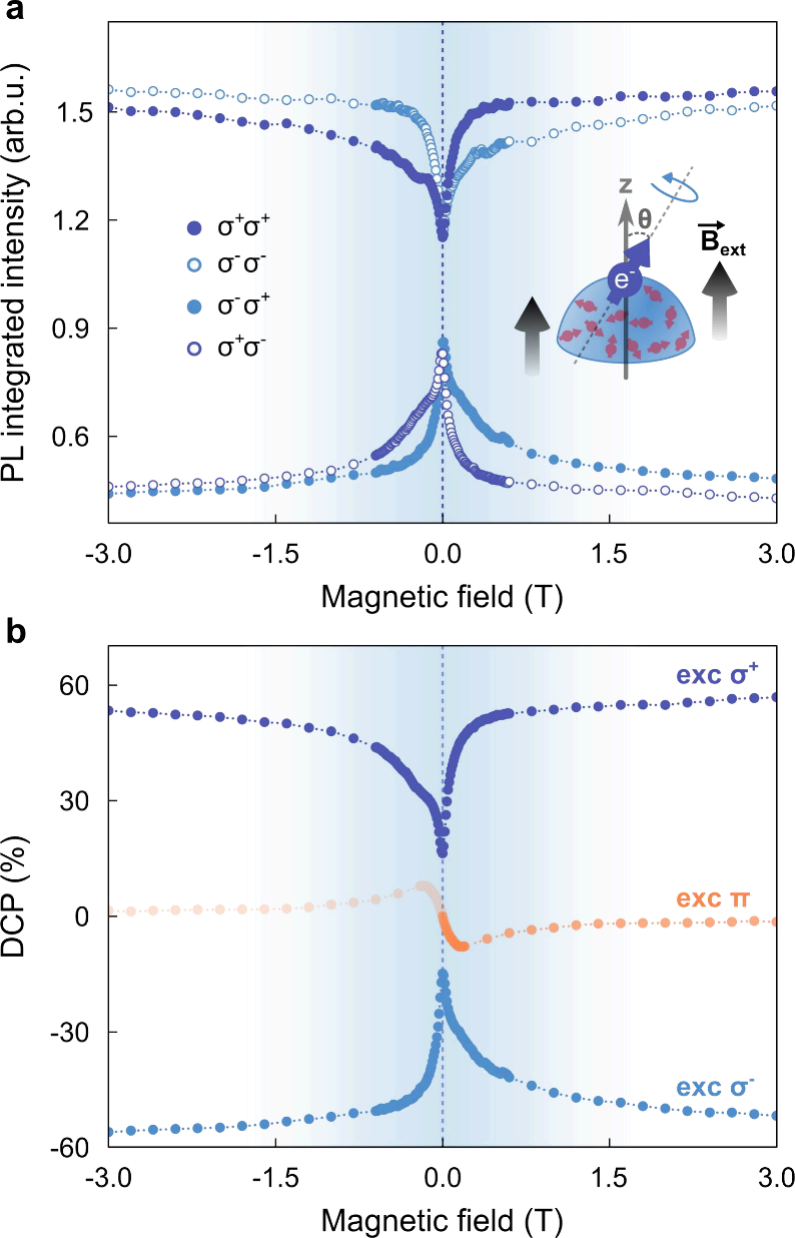
Effects of hyperfine interaction on the low-magnetic-field optical polarization in the type-II QDs. (a) PL integrated intensity as a function of the external magnetic field under circularly polarized excitations (σ^+^ and σ^–^) at an excitation density of 7800 W/cm^2^. The inset represents the electron spin precession around an effective magnetic field tilted from the direction (*z*) of the external stimulus due to the interaction with the nuclear spin bath. (b) Degrees of circular polarization (DCP) under linearly and circularly polarized incident light. For the linear case, the branch at negative magnetic fields is shown as translucent, as it is merely a mirror reflection of the measured positive-field data.

At 0 T, the DCP reaches average values of ±15.6% for σ^+^ and σ^–^ excitation. When a magnetic field of 0.2 T is applied, the sharper side of the DCP curves exhibits a substantial enhancement, with values of 47.0% and −44.7% for σ^+^ and σ^–^ excitation, respectively. This 3-fold increase is a hallmark of electron spin precession in the presence of frozen nuclear spin fluctuations mediated by the hyperfine interaction.^65–67^ A schematic of this process is shown in the inset of Figure 3a. For reference, the DCP obtained under linearly polarized (π) excitation is also included in Figure 3b.

Additionally, the DCP recovery curves display a pronounced helicity-dependent asymmetry: under σ^+^ excitation, the DCP recovers more slowly for negative fields, whereas the opposite trend occurs for σ^–^ excitation. This asymmetric behavior indicates the presence of a field-dependent contribution to the nuclear spin polarization.^68^ In our system, these contributions arise because the exciton populations—and therefore the average electron spin that polarizes the nuclei via the Knight field—vary with magnetic field through both LAC-induced bright–dark mixing and thermal redistribution among the fine-structure states. Consequently, the effective nuclear field acquires a weak field dependence, resulting in the asymmetric DCP profile observed in Figure 3b.

The influence of hyperfine fields on hole spins is significantly suppressed due to both p-type character of the Bloch wave function and delocalization in the GaAsSb layer.^9,69^ This explains why only a single LAC-induced-PL feature is observed and why the anticrossing involves bright and dark states with the same heavy-hole spin projection, consistent with the proposed exciton fine structure.

It is worth noting that in-plane spin precession has been previously associated with reduced QD rotational symmetry.^21^ However, AFM images of uncapped samples (Figure S3) show weak in-plane elongation in our QDs, which is further protected by the capping process with the GaAs_1–*x*_Sb_*x*_ strain-reducing layer.^47^ We also emphasize that, despite similar polarization profiles, our findings are not related to the recently proposed mechanism of dynamic electron spin polarization,^9^ as discussed in the Supporting Information.

In summary, we demonstrated the emergence of single-handed circularly polarized light (19%) at *B*_*c*_ ∼ 0.17 T in type-II In­(Ga)­As/GaAsSb QDs as a consequence of level anticrossing and bright–dark mixing. The effect is driven by the electron–nuclear spin interaction, which introduces an in-plane effective magnetic field component in the spin Hamiltonian. The weak field originates from the suppressed electron–hole exchange interaction due to spatial carrier separation in the type-II architecture. By developing a population model for the spin-split states, experimental PL intensities and polarization degrees are successfully reproduced, showing that the mirror-symmetric response of the distinct-helicity emissions stems from spin lifetimes that are finite yet longer than the optical recombination time as well as the presence of long-lived dark states. Optical orientation measurements and AFM analysis confirm the role of hyperfine interaction in inducing LAC, while ruling out structural anisotropy. The data verify that the mixing involves only one pair of bright and dark states, leading to a single, time-independent LAC-induced polarization peak, which is resonantly tunable within a narrow range of weak magnetic fields. Together with the inherently high initialization fidelity,^16^ our findings establish type-II quantum dots as promising emitters of circularly polarized light, operable under nonresonant, linearly polarized excitation and weak magnetic fields, opening new pathways for compact, integrated spin-optoelectronic devices.

## Supplementary Material



## Data Availability

Data for this article, including the PL spectra as a function of the external magnetic field, time-resolved PL, power-dependent-PL, electronic structure simulations, AFM images, and the code used for the theoretical framework are available on Open Science Framework at 10.17605/OSF.IO/UP6R8.

## Notes

Description of the samples’ growth and experimental methods; description and additional analysis from the electronic structure simulations; development of the theoretical framework employed; magneto-PL characterization under different excitation densities and effective g-factor estimation; structural characterization of the QD ensemble (AFM images); comparison between LAC-induced and dynamic electron spin polarization; and discussion on charged QD states (PDF)
