# Supplementary material for: Low-Field Optical Polarization in Type-II Quantum Dots via Nuclear-Driven Dark State Mixing
Source: Nano Lett. 2025 Dec 22;26(1):314–21. doi: 10.1021/acs.nanolett.5c05163 (PMC12810464; doi:10.1021/acs.nanolett.5c05163)
Supplement: Supplementary file 1 [file nl5c05163_si_001.pdf]

SUPPORTING INFORMATION: Low-Field Optical Polarization in Type-II Quantum Dots via Nuclear-Driven Dark State Mixing

Gabriel M. Jacobsen,[1,2] Vinicius A. de Oliveira,[1] Baolai Liang,[3] Morgan E. Ware,[2] Gregory J. Salamo,[2] Gilmar E. Marques,[1] Yuriy I. Mazur,[2] Victor Lopez-Richard,[1] and Marcio D. Teodoro[1,*]

[1]*Department of Physics, Federal University of São Carlos, 13565-905, São Carlos, São Paulo, Brazil*
[2]*Institute for Nanoscience and Engineering, University of Arkansas, 72701, Fayetteville, Arkansas, USA*
[3]*Department of Electrical and Computer Engineering, California NanoSystems Institute, University of California - Los Angeles, 90095, Los Angeles, California, USA*

Supporting information 1 | Sample growth and experimental methods

The In(Ga)As/GaAs$_{1-x}$Sb$_x$ QD samples were grown by molecular beam epitaxy with a Veeco Gen-930 reactor.[1] The growth started by depositing a 100 nm GaAs buffer layer at 580 °C on top of a semi-insulating GaAs (100) substrate. Next, 2 monolayers (MLs) of InAs were deposited at 525 °C with a growth rate of 0.015 ML/s. Then, the QDs were capped by a 10-nm layer of GaAs$_{1-x}$Sb$_x$ with x equal to 11% and 15%, yielding the type-I and type-II band alignments, respectively.[2,3] Finally, a 60 nm GaAs layer was added to complete the structure. According to the AFM images from uncapped samples, the lens-shaped QD ensemble has an areal density of 2.6×10^{10} cm^{-2} and shows an average diameter of 54.5 ± 6.3 nm and an average height of 13.6 ± 2.4 nm.

For the magneto-photoluminescence measurements, the samples were loaded inside a helium closed-cycle cryostat (Attocube/Attodry 1000) with a base temperature of 3.7 K and equipped with a superconductor solenoid capable of generating magnetic fields up to ±9 T in the Faraday geometry (magnetic field applied parallel to the sample's growth axis). A low-temperature aspheric objective (NA = 0.68) focused the laser beam on a spot of ≈ 1 μm. For the level anticrossing investigation, a linearly polarized 730 nm (1.698 eV) pulsed diode laser (PicoQuant/LDH-730) with a repetition rate of 20 MHz was employed. For detection, a quarter-wave plate and a Glan-Taylor polarizer were used to convert the emitted circularly polarized light, represented by the σ^+ and σ^- components, to the linear basis. On the other hand, for the polarization recovery studies, the circularly polarized emission from a 1080 nm (1.148 eV) pulsed diode laser (PicoQuant/LDH-1080) was employed, while the detection arm used a half-wave plate and a Glan-Taylor polarizer to separate the σ^+ and σ^- luminescence. The PL signal was dispersed by a 50 cm spectrometer and detected by an InGaAs diode array detector (Andor/Shamrock-Idus). The transient measurements were performed using a near-infrared photomultiplier tube unit (Hamamatsu/NIR-PMT) connected to a standard TCSPC electronics (PicoHarp300).

Supporting information 2 | Electronic structure simulations

Numeric simulations of the 3D electronic structure of the QDs were performed with the Nextnano software.[4] The QD structure consists of a lens-shaped In$_{1-y}$Ga$_y$As QD, whose diameter and height were defined by the average values extracted from the AFM analysis, lying on top of a 0.6 nm wetting layer (matching the 2 MLs deposited during growth). Diffusion and segregation of In, Sb, and Ga atoms play an important role in InAs/GaAsSb structures, leading to the formation of a ternary alloy in the QD region along with different possibilities for the composition profiles.[5–7]. Following the approach in Ref. 8, we use the Ga content to match the optical transition energy obtained in the PL measurements, yielding $y = 40\%$ and 28% for the type-I and type-II QDs, respectively, that agree well to the value determined by sectional scanning tunneling microscopy (X-STM) in the same reference. For simplicity, we considered a constant compositional profile inside the QD. Additionally, reduction of QD dimensions after capping is a typical feature in InAs/GaAs systems.[9] Here, however, due to the surfactant effect of Sb and the reduced strain owed to the GaAsSb layer, a progressive suppression of the QD decomposition with Sb content is observed.[10] Hence, for our cases with [Sb] = 11% or 15%, we used the average height from the AFM of the uncapped QDs weighted by a scaling factor estimated from Ref. 10: 0.7 for 11% and 1.0 for 15% and above.

After initializing the structure, the strain is taken into account by applying the continuum elastic model, suitable for QDs,[11] along with the corresponding linear piezoelectric field and the following solution of the Poisson equation. Then, the energy levels and wavefunctions are solved self-consistently inside a box ranging 10 nm of all QD limits by using an 8-band $\mathbf{k}\cdot\mathbf{p}$ model for the Schrödinger equation where Dirichlet boundary conditions were assumed. A step size of 1 nm was considered in the QD plane, while a finer grid (up to 0.1 nm) was employed along the growth direction. All of the parameters used in the calculations were taken from the Nextnano database and Ref. 12.

* mdaldin@ufscar.br

Here, we present additional data supporting our discussion and results. Figure S1a shows a 2D (x-z plane) energy colormap of the heavy-hole (HH) band for $x = 15\%$. The dark-bluish regions correspond to the HH energy absolute minima, located at the lateral interfaces between the QD and GaAsSb layers, consistent with the 2D probability map shown in Figure 1b of the main text.

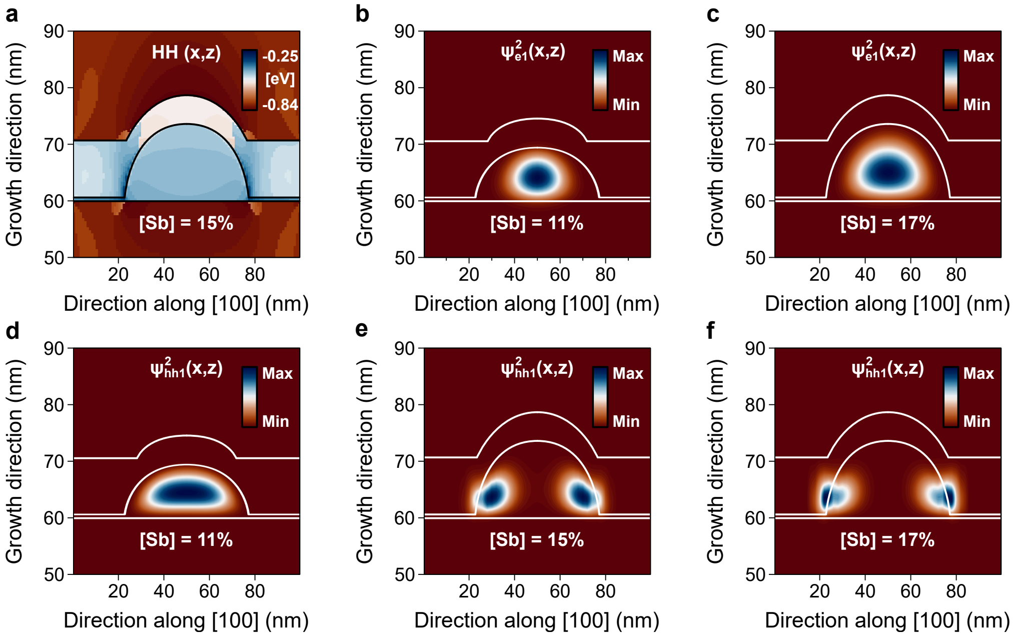

Figure S1. (a) 2D colormap for the heavy-hole (HH) band for the type-II QD (In(Ga)As/$GaAs_{0.85}Sb_{0.15}$). 2D probability density colormap for the fundamental electron subband for [Sb] in the GaAsSb layer equal to (b) 11% and (c) 17%. 2D probability density colormap for the fundamental heavy-hole subband for [Sb] in the GaAsSb layer equal to (d) 11%, (e) 15%, and (f) 17%. Lines delineating the QD and GaAsSb regions are shown to guide the reader.

Figure S1b–f presents a series of 2D probability colormaps of the fundamental electron and heavy-hole subbands for nominal Sb compositions of 11%, 15%, and 17% in the GaAsSb layer. The different QD heights observed for x equal to 11% compared to the higher Sb contents — outlined by the white interface lines — reflect the estimated reduction due to the capping process, while the dot diameter remains unchanged.[10]

The electron probability density (Ψ^2_{e1}) is only weakly affected by changes in the GaAsSb composition. Thus, we show only the results for [Sb] equal to 11% and 17% in Figure S1b,c. In contrast, the heavy-hole probability density (Ψ^2_{hh1}) exhibits substantial variation with increasing Sb content (Figure S1d–f). As [Sb] increases, Ψ^2_{hh1} splits into two lobes that migrate toward the QD rim due to inhomogeneous strain and piezoelectric fields, ultimately inducing a ring-like topology in the wavefunction probability density.[8,13,14] This leads to a strong reduction in the electron-hole wavefunction overlap for x above 11%, consistent with the long optical recombination time of 17.3 ns observed for the sample with [Sb] equal to 15% and the one-order shift in the level anticrossing (LAC) positions between type-I and type-II QDs, attributable to the weakened short-range electron-hole exchange interaction.[15–17]

Supporting information 3 | Spin Hamiltonian and population model

The fine structure of excitons is defined by the spin Hamiltonian ($\mathcal{H}_s$),[18–20] which includes the electron-hole exchange interaction ($\mathcal{H}_{exch}$) and the Zeeman splitting ($\mathcal{H}_Z$). To enable the emergence of anticrossings (hybridization) between the bright and dark states, off-diagonal elements must be introduced via a perturbation potential ($V_{ii'}$). Then, the spin Hamiltonian reads as:

$$\mathcal{H}_s = \mathcal{H}_{exch} + \mathcal{H}_Z + V_{ii'}, \tag{1}$$

$$\mathcal{H}_s = \frac{1}{2}\begin{bmatrix} \delta_0 + \mu_B B(-g_e^z + g_h^z) & \delta_b & V_{13} & V_{14} \\ \delta_b & \delta_0 + \mu_B B(g_e^z - g_h^z) & V_{23} & V_{24} \\ V_{31} & V_{32} & -\delta_0 + \mu_B B(-g_e^z - g_h^z) & \delta_d \\ V_{41} & V_{42} & \delta_d & -\delta_0 + \mu_B B(g_e^z + g_h^z) \end{bmatrix}, \tag{2}$$

where δ_0, δ_b, and δ_d represent the exchange splittings between bright and dark states, μ_B is the Bohr magneton, B is the strength of the external magnetic field along the growth direction (z), and $g^z_{e(h)}$ is the electron (heavy-hole) out-of-plane g-factor. We also consider symmetric off-diagonal elements, such that $V_{ii'} = V_{i'i}$.

The four-level model is represented by $|i\rangle = \sum_m C_{i,m} |m\rangle_z$, where $i = 1, 2, 3, 4$ references each one of the four states in the total-angular-momentum basis set, $|m\rangle_z$, of the bright and dark states ($|\pm1\rangle$ and $|\pm2\rangle$). The eigenenergies (E_i) and projections ($C_{i,m}$) obtained from solving the Hamiltonian feed back into the electron-hole population (f_i) described by the following set of rate equations:

$$\frac{\partial f_i}{\partial t} = -\frac{1}{\tau_i} f_i - \sum_{i' \neq i} \left(\frac{f_i}{T_{i'i}} - \frac{f_{i'}}{T_{ii'}} \right) + G_i = 0, \tag{3}$$

where

$$\frac{1}{\tau_i} = \frac{1}{\tau_r} (|C_{i,1}|^2 + |C_{i,-1}|^2) + \frac{1}{\tau_0}, \tag{4}$$

$$\frac{1}{T_{i'i}} = \frac{w_{i'i}}{\tau_s} \sum_{m'm} \eta_{m'm} |C_{i,m} C_{i',m'}|^2. \tag{5}$$

The terms τ_r and τ_0 represent the radiative (optical) and non-radiative recombination times. The transfer rate between the levels, $T^{-1}_{ii'}$, is weighed by the spin-flip time (τ_s) and a thermal activation factor given by $w_{i'i}$ =exp$[(E_i - E_{i'})/(k_B T)]$, where k_B is the Boltzmann constant and T is the effective temperature of the system. For an accurate description, we estimate the latter by fitting the high-energy tail of the PL spectrum with a Boltzmann-like function,[21] extracting $T \approx 10$ K, slightly above the lattice temperature of 3.7 K. The parameter $\eta_{m'm}$ defines whether electrons or holes are allowed to spin-flip. In our case, both mechanisms are considered.

Finally, the rate equations also incorporate carrier generation terms (G_i) defined by:

$$G_i = G_r \left| \sum_m C_{i,m} M_m \right|^2 + G_0, \tag{6}$$

where G_r and G_0 are the generation terms for bright and dark states, respectively, and M_m is equal to unity for $m = \pm 1$, and zero otherwise. For the case of non-resonant excitation, the condition of $G_0 \gg G_r$ is typically observed.

For simplicity, we reduced the four-level system to three levels, considering both bright states ($|\pm1\rangle$) and only the dark state that anticrosses a bright one, which is $|-2\rangle$. In other words, as V_{24} is negligible as a result of the perturbation coming from the hyperfine interaction between the electron and nuclear spins, and the fact that E_1 never crosses (or anticrosses) E_4, the influence of the dark state $|+2\rangle$ in the spin dynamics is insignificant and thus, the spin Hamiltonian can be reduced to a 3×3 matrix.

Supporting information 4 | Magneto-PL under different excitation densities for the type-II QD

In the main text, we presented PL colormaps and DCP curves for the type-II QDs under varying excitation densities, focusing on the low-magnetic-field regime. For completeness, Figure S2a–c provides (i) the type-II QD normalized PL spectra at zero field for all the excitation densities used, (ii) the Zeeman splitting used to extract the electron-hole pair g-factor, and (iii) the degree of circular polarization over the full magnetic field range (0 to 9 T).

With a three-order increase in excitation density, the PL peak exhibits a 31 meV blueshift in Figure S2a. This shift is characteristic of type-II systems, where spatial carrier separation induces the formation of an electric dipole that bends the conduction and valence bands, leading to a $P^{1/3}_{exc}$ dependence of the emission energy.[22,23] The higher excitation also enables the population of higher-energy levels, resulting in the appearance of an additional peak at 3000 W/cm^2, attributed to the QD excited states.[1]

In the context of the fine structure and magnetic response of electron-hole pairs in QDs, the Landé g-factor plays a critical role, as can be seen in the spin Hamiltonian framework developed here that depends on the individual out-of-plane electron and heavy-hole g-factors. However, magneto-PL measurements in the Faraday geometry only provide information about the out-of-plane electron-hole pair g-factor, g^z_{e-h}. To solve this issue, we chose to estimate the out-of-plane electron g-factor value (g^z_e) according to the extensive calculations from Ref. 24, leading to g^z_e equal to -2.4 and then, we use it to calculate the out-of-plane heavy-hole g-factor given by the sum between g^z_e and g^z_{e-h}

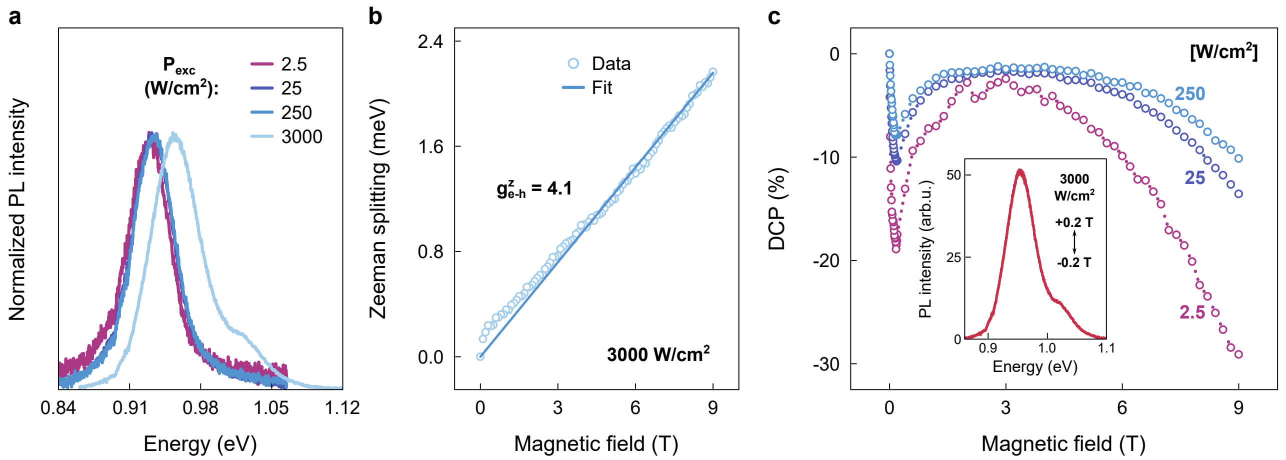

Figure S2. (a) Normalized PL spectra for the type-II $InAs/GaAs_{0.85}Sb_{0.15}$ QD at 3.7 K and different excitation power densities: 2.5, 25, 250, and 3000 W/cm^2. (b) Zeeman splitting of the type-II optical transition measured at 3000 W/cm^2, with a linear fit used to extract the electron-hole pair g-factor. (c) Degrees of circular polarization (DCP) with distinct excitation densities. The inset shows the PL spectra at 0, ±0.1, and ±0.2 T with 3000 W/cm^2.

($g^z_{e-h} = g^z_h - g^z_e$). From the linear fit ($E_{\sigma^+} - E_{\sigma^-} = \mu_B g^z_{e-h} B$) of the Zeeman splitting in Figure S2b, we obtain $g^z_{e-h} = 4.1$, yielding $g^z_h = 1.7$.

Finally, we display the DCP curves for 2.5, 25, and 250 W/cm^2 over the 0–9 T range in Figure S2c. The inset includes five PL spectra at 3000 W/cm^2 and magnetic fields of 0, ±0.1, and ±0.2 T. The results indicate that the anticrossing involves the lower-energy bright state and the higher-energy dark state, since the LAC polarization sign matches the one from the thermal contribution at high magnetic fields. Additionally, the positive Zeeman splitting ($g^z_{e-h} > 0$) reveals that the lowest bright state correspond to the σ^- ($|-1\rangle$) component. As hyperfine-induced (interaction between electron and nuclear spins) bright-dark state mixing requires identical heavy-hole spin projections,[25] we conclude that the LAC occurs between the $|-1\rangle$ (bright) and $|-2\rangle$ (dark) states, which agrees with our model and g-factor assignment (see the 2D eigenenergy colormap in Figure 2a in the main text).

We also observe a strong reduction in LAC polarization amplitude with increasing excitation density, where total suppression occurs at 3000 W/cm^2 as demonstrated by the unchanged PL spectral up to ±0.2 T in the inset of Figure S2c. Our simulations show that the LAC amplitude is governed by the ratio of bright-to-dark generation rates (G_r/G_0) and by the system's characteristic times (τ_r, τ_0, and τ_s), being temperature independent. Hence, we attribute the amplitude reduction to a decrease in spin-flip time, τ_s, caused by the elevated effective temperature at high excitation densities.[17,26] Furthermore, thermal polarization, observed at high magnetic fields, decreases in higher temperatures due to the activation factor $w_{i'i} = \exp[(E_i - E_{i'})/(k_B T)]$. We note that, while faster spin-flip rates (smaller τ_s) tend to enhance thermal polarization, the overall DCP decrease with higher excitation density implies that direct temperature dependence via $w_{i'i}$ dominates in this regime. In addition, we verified that the use of the 1.148 eV excitation line at high excitation density (7800 W/cm^2) — employed in optical orientation (polarization recovery) experiments — still permitted the observation of LAC effects. In this case, excitation below the GaAs bandgap results in a significantly reduced light absorption efficiency compared to the 1.698 eV excitation, where similar PL intensities are obtained for the excitation densities of 25 and 7800 W/cm^2 at 1.698 and 1.148 eV, respectively.

Supporting information 5 | Structural characterization of QD ensemble

Figure S3a displays a 2×2 μm AFM image of uncapped QDs. Visually, the dots ensemble is formed by islands with high in-plane (x-y) symmetry. To confirm this, we extract horizontal (x) and vertical (y) profiles for five representative QDs, as depicted in Figure S3b,c.

Reduction of the rotational symmetry in dot structures significantly affects the electron-hole fine structure, influencing lateral wavefunction distribution and the long-range contribution of the exchange interaction. Thus, it is directly linked to the fine structure splitting between bright states, represented by δ_b in our theoretical framework, and even responsible for introducing off-diagonal elements in the spin Hamiltonian.[13,16,20]

By combining the QD profiles along the [100] and [010] directions depicted in Figure S3 and experimental evidences of unchanged QD base length with the capping process in InAs/GaAsSb QDs from Ref. 10, we conclude that our type-I and type-II QD samples have weak in-plane ellipticity. This supports our decision to neglect δ_b in our simulations,

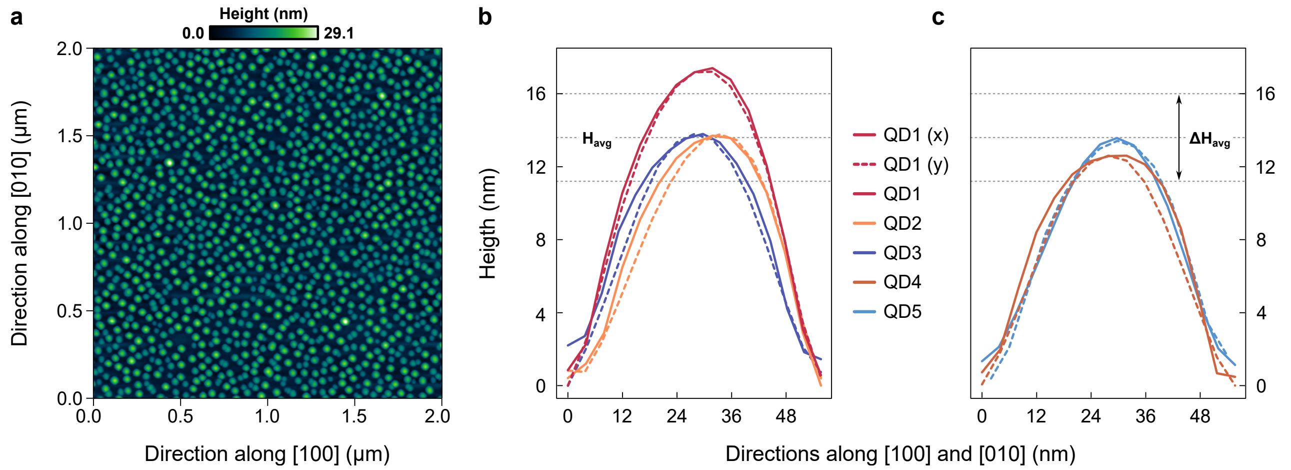

Figure S3. (a) 2×2 μm AFM image of uncapped QDs. (b, c) Representative QD profiles along the [100] and [010] directions.

as its estimated values obtained from theoretical studies in InAs/GaAsSb QDs in Ref. 13 (≈-10 to 10 μeV) do not impact the LAC features. Furthermore, the weak reduction in the QDs symmetry (D_2d) implicate that the primary ingredient of the perturbation in the spin Hamiltonian of our QD system is the hyperfine interaction.

Supporting information 6 | Level anticrossing and dynamic electron spin polarization

A recent report[17] has identified a distinct mechanism capable of generating single-handed circularly polarized emission from quantum dots under weak magnetic fields: the dynamic electron spin polarization (DEP). Because DEP can produce magnetic-field dependences that resemble, at least qualitatively, those arising from level anticrossing (LAC) and bright–dark exciton hybridization, it is essential to examine both mechanisms side by side. This comparison serves to clarify their fundamental differences and to highlight the unique microscopic origin of the polarization observed in our type-II QDs.

To this end, we first outline the steady-state formalism describing the DEP mechanism. In a QD system where electrons precess around a total magnetic field composed of the external field B (Faraday geometry), the exchange field B_{exch}, and the effective nuclear field B_N, the total populations N^+ (N^-) of the exciton states with total angular momentum +1 and +2 (−1 and −2), together with their corresponding longitudinal electron-spin components $S_{\parallel}^{\pm}$, evolve according to

$$\frac{dN^{\pm}}{dt} = \frac{G}{2} - \frac{N^{\pm}}{2\tau_b} \pm \frac{S_{\parallel}^{\pm}\cos(\phi_{\pm})}{\tau_b}, \tag{7}$$

and

$$\frac{dS_{\parallel}^{\pm}}{dt} = -\frac{S_{\parallel}^{\pm}}{2\tau_b} \pm \frac{N^{\pm}\cos(\phi_{\pm})}{4\tau_b}. \tag{8}$$

Here, G is a generation rate, τ_b is the bright exciton lifetime, and $\phi_{\pm}$ are the angles between the total magnetic field and the quantization axis (z)

$$\mathbf{B_{tot}^{\pm} e_z} = B + B_N^z \pm B_{exch} = B_{tot}^{\pm}\cos(\phi_{\pm}), \tag{9}$$

where $\mathbf{e_z}$ is the unit vector along z and B_N^z is the effective nuclear field along the quantization axis. In addition, $N^+ + N^- = N_b + N_d$, where N_b and N_d are the numbers of bright and dark excitons, respectively, and the longitudinal electron spin in the z axis is

$$\langle S_z \rangle = S_z^+ + S_z^-, \tag{10}$$

with

$$S_z^{\pm} = S_{\parallel}^{\pm} cos(\phi_{\pm}). \tag{11}$$

In turn, the intensities of the circularly polarized components, σ^+ and σ^-, are described by

$$I_{\sigma^\pm} \propto \left\langle \frac{N_b}{2} \pm \frac{1}{2}\left(\frac{J_z}{3} - S_z\right)\right\rangle \frac{1}{\tau_r}, \tag{12}$$

where J_z is the heavy-hole spin ($\pm 3/2$) and τ_r is the radiative recombination time. Thus, averaging the nuclear field over a Gaussian distribution with dispersion Δ_B, the circularly polarized intensities follow

$$I_{\sigma^\pm} \propto \frac{\tau_b G}{2\tau_r}\left[\frac{2(B \mp B_{exch})^2 + \Delta_B^2/2}{\Delta_B^2/2}\right], \tag{13}$$

leading, in the steady state, to the DCP expression

$$DCP = \frac{I_{\sigma^+} - I_{\sigma^-}}{I_{\sigma^+} + I_{\sigma^-}} = -\frac{2BB_{exch}}{B^2 + B_{exch}^2 + \Delta_B^2/4}. \tag{14}$$

The comparison between DEP and the LAC model used in our work is shown in Figure S4. For the DEP curves we used $\tau_b/\tau_r = 1$, $G = 1$, $B_{exch} = 0.122$ T, and $\Delta_B = 0.218$ T, chosen to achieve the best possible match to the LAC-induced polarization amplitude and position. The orange curves, corresponding to the LAC model, are taken from Figures 2e,f of the main text.

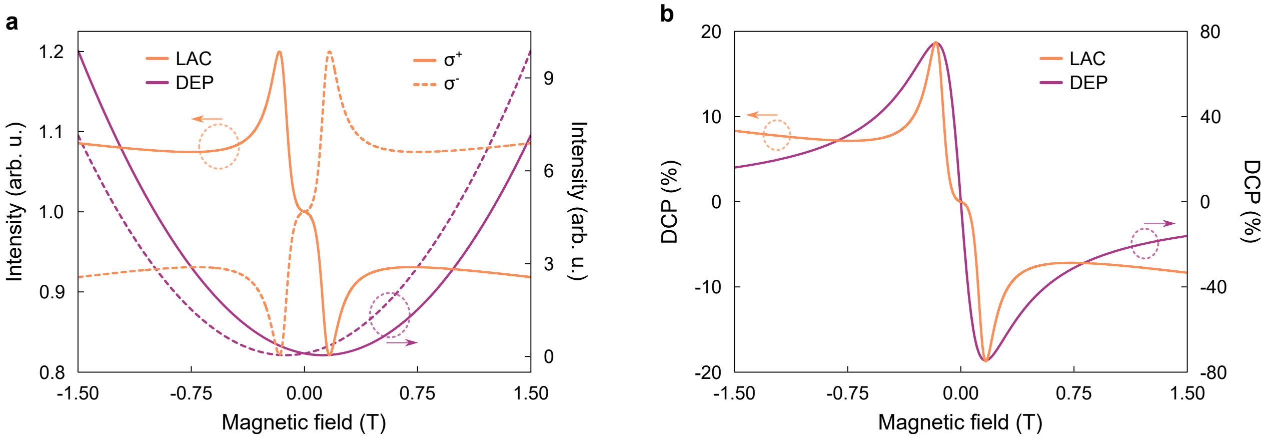

Figure S4. (a) Integrated intensities from the circularly polarized components, σ^+ and σ^-, and (b) degree of circular polarization (DCP) with the level anticrossing (LAC) and dynamic electron spin polarization (DEP) models.

Our analysis confirms that the magnetic-field dependence of the DCP alone can indeed appear similar for both mechanisms (Figure S4b) despite the monotonic DEP decrease after the maximum. However, a decisive difference emerges: the individual intensity traces $I_{\sigma^+}(B)$ and $I_{\sigma^-}(B)$, exhibit qualitatively different behavior in the two mechanisms as depicted in Figure S4a.

In DEP, polarization arises from nuclei-assisted spin flips and consequently, bright-dark exciton mixing which modifies the exciton species. In contrast, in our type-II QDs, the dominant mechanism is the direct hybridization of bright and dark states via level anticrossing, which produces a sharp resonance enhancement in one bright-exciton line and, due to the particular carrier and spin dynamics of our type-II QDs, a simultaneous valley in the other. These contrasting intensity patterns provide an unambiguous fingerprint distinguishing the two mechanisms. This distinction forms the basis of the originality of our results.

Supporting information 7 | Discussion on charged QD states

In our analysis, we assume that the QDs are predominantly neutral and neglect contributions from charged excitonic complexes. This assumption is supported by several observations. First, in undoped, high-density (10^{10} cm^{-2}) QDs ensembles, the spontaneous formation of charged excitons is highly unlikely.[27] Nonetheless, in the exceptional case where residual unintentional doping is sufficient to charge the QDs, additional optical transitions from trions or biexcitons would emerge, as previously reported in Ref. 28. Such complexes typically produce pronounced changes in PL spectra as a function of excitation power and temperature due to their distinct carrier dynamics.

As shown in Fig. S2a and in the complementary power- and temperature-dependent studies on the same type-I and type-II QD systems (Ref. 1), the PL spectra remain dominated by a single Gaussian band across nearly four

orders of magnitude in excitation density and from 10 to 150 K. Aside from the expected blueshift associated with type-II confinement and the appearance of excited-state emission at high excitation densities, no features indicative of charged complexes are observed.

To further confirm the neutral nature of the emission, we analyze the power dependence of the PL integrated intensity in the type-II QDs (Fig. S5). Fitting the data with $I \propto P^{\gamma}$ yields $\gamma_1 = 0.984$ up to $\sim$200 W/cm^2, which is characteristic of neutral-exciton recombination and excludes biexciton contributions.[28] At higher excitation densities, the exponent decreases to $\gamma_2 = 0.772$, a behavior consistent with state saturation and with the high-energy excited-state bands visible in Fig. S2a.

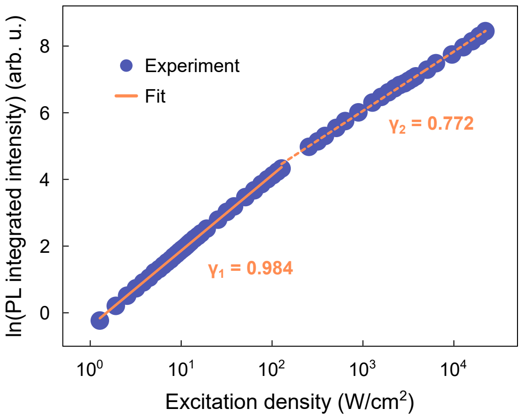

Figure S5. ln of the PL integrated intensity from the type-II QD as a function of the excitation density with fits (solid line) by a $I \propto P^{\gamma}$ function, where γ can be linked to the nature of the optical recombination.

Finally, we note that in InAs/GaAs-based QDs, the lowest trion state is typically the singlet trion, for which the electron–hole exchange interaction vanishes.[29] Since level anticrossing requires a finite bright–dark exchange splitting, trions cannot participate in or generate the LAC-induced polarization resonance. This conclusion is consistent with Fig. 2d, where all QDs emitting within a ±32 meV window around the type-II PL peak exhibit identical LAC-induced DCP behavior, something incompatible with a mixture of neutral and charged emitters.

For completeness, we remark that carrier exchange between neutral and trion states has been proposed as a potential route to achieve 100% circularly polarized emission in electrically injected QDs, relevant to spin-LED architectures.[25] Such scenarios require intentional n- and p-type contacts and therefore lie beyond the scope of the present work. Nonetheless, we highlight this possibility to motivate future investigations of LAC-driven polarization phenomena in electrically charged type-II QD systems.

[1] C. Zhou, B. Liang, J. Liu, Y. Wang, Y. Guo, S. Wang, G. Fu, Yu. I. Mazur, M. E. Ware, and G. J. Salamo, Comparative study of photoluminescence for type-I InAs/GaAs$_{0.89}$Sb$_{0.11}$ and type-II InAs/GaAs$_{0.85}$Sb$_{0.15}$ quantum dots, Opt. Mater. (Amst.) **98**, 109479 (2019).

[2] D. Kim, S. Hatch, J. Wu, K. A. Sablon, P. Lam, P. Jurczak, M. Tang, W. P. Gillin, and H. Liu, Type-II InAs/GaAsSb Quantum Dot Solar Cells With GaAs Interlayer, IEEE J. Photovoltaics **8**, 741 (2018).

[3] T. T. Chen, C. L. Cheng, Y. F. Chen, F. Y. Chang, H. H. Lin, C.-T. Wu, and C.-H. Chen, Unusual optical properties of type-II InAs/GaAs$_{0.7}$Sb$_{0.3}$ quantum dots by photoluminescence studies, Phys. Rev. B **75**, 033310 (2007).

[4] S. Birner, T. Zibold, T. Andlauer, T. Kubis, M. Sabathil, A. Trellakis, and P. Vogl, nextnano: General Purpose 3-D Simulations, IEEE Trans. Electron Devices **54**, 2137 (2007).

[5] P. Klenovský, V. Křápek, D. Munzar, and J. Humlíček, Electronic structure of InAs quantum dots with GaAsSb strain reducing layer: Localization of holes and its effect on the optical properties, Appl. Phys. Lett. **97**, 203107 (2010).

[6] V. Haxha, I. Drouzas, J. M. Ulloa, M. Bozkurt, P. M. Koenraad, D. J. Mowbray, H. Y. Liu, M. J. Steer, M. Hopkinson, and M. A. Migliorato, Role of segregation in InAs/GaAs quantum dot structures capped with a GaAsSb strain-reduction layer, Phys. Rev. B **80**, 165334 (2009).

[7] P. Klenovský, D. Hemzal, P. Steindl, M. Zíková, V. Křápek, and J. Humlíček, Polarization anisotropy of the emission from type-II quantum dots, Phys. Rev. B **92**, 241302(R) (2015).

[8] J. M. Ulloa, J. M. Llorens, M. del Moral, M. Bozkurt, P. M. Koenraad, and A. Hierro, Analysis of the modified optical properties and band structure of GaAs$_{1-x}$Sb$_x$-capped InAs/GaAs quantum dots, J. Appl. Phys. **112**, 074311 (2012).

[9] Q. Gong, P. Offermans, R. Nötzel, P. M. Koenraad, and J. H. Wolter, Capping process of InAs/GaAs quantum dots studied by cross-sectional scanning tunneling microscopy, Appl. Phys. Lett. **85**, 5697 (2004).
[10] J. M. Ulloa, R. Gargallo-Caballero, M. Bozkurt, M. Del Moral, A. Guzmán, P. M. Koenraad, and A. Hierro, GaAsSb-capped InAs quantum dots: From enlarged quantum dot height to alloy fluctuations, Phys. Rev. B **81**, 165305 (2010).
[11] A. Mittelstädt, A. Schliwa, and P. Klenovský, Modeling electronic and optical properties of III–V quantum dots—selected recent developments, Light Sci. Appl. **11**, 17 (2022).
[12] I. Vurgaftman, J. Meyer, and R. Ram-Mohan, Band parameters for III-V compound semiconductors and their alloys, J. Appl. Phys. **89**, 5815 (2001).
[13] V. Křápek, P. Klenovský, and T. Šikola, Excitonic fine structure splitting in type-II quantum dots, Phys. Rev. B **92**, 195430 (2015).
[14] J. M. Llorens, V. Lopes-Oliveira, V. Lopez-Richard, E. R. C. de Oliveira, L. Wewiór, J. M. Ulloa, M. D. Teodoro, G. E. Marques, A. García-Cristóbal, G.-Q. Hai, and B. Alén, Topology Driven g-Factor Tuning in Type-II Quantum Dots, Phys. Rev. Appl. **11**, 044011 (2019).
[15] E. Blackwood, M. J. Snelling, R. T. Harley, S. R. Andrews, and C. T. B. Foxon, Exchange interaction of excitons in GaAs heterostructures, Phys. Rev. B **50**, 14246 (1994).
[16] M. Bayer, G. Ortner, O. Stern, A. Kuther, A. A. Gorbunov, A. Forchel, P. Hawrylak, S. Fafard, K. Hinzer, T. L. Reinecke, S. N. Walck, J. P. Reithmaier, F. Klopf, and F. Schäfer, Fine structure of neutral and charged excitons in self-assembled In(Ga)As/(Al)GaAs quantum dots, Phys. Rev. B **65**, 195315 (2002).
[17] D. S. Smirnov, T. S. Shamirzaev, D. R. Yakovlev, and M. Bayer, Dynamic Polarization of Electron Spins Interacting with Nuclei in Semiconductor Nanostructures, Phys. Rev. Lett. **125**, 156801 (2020).
[18] E. L. Ivchenko, Spectroscopy of spin-polarized excitons in semiconductors, Pure & Appl. Chem. **67**, 463 (1995).
[19] M. Bayer, O. Stern, A. Kuther, and A. Forchel, Spectroscopic study of dark excitons in $In_xGa_{1-x}As$ self-assembled quantum dots by a magnetic-field-induced symmetry breaking, Phys. Rev. B **61**, 7273 (2000).
[20] Y. Masumoto, K. Toshiyuki, T. Suzuki, and M. Ikezawa, Resonant spin orientation at the exciton level anticrossing in InP quantum dots, Phys. Rev. B **77**, 115331 (2008).
[21] V. Laurindo Jr., E. D. Guarin Castro, G. M. Jacobsen, E. R. C. de Oliveira, J. F. M. Domenegueti, B. Alén, Y. I. Mazur, G. J. Salamo, G. E. Marques, E. Marega Jr., M. D. Teodoro, and V. Lopez-Richard, Spin-dependent analysis of homogeneous and inhomogeneous exciton decoherence in magnetic fields, Phys. Rev. B **105**, 045414 (2022).
[22] D. S. Abramkin, A. K. Gutakovskii, and T. S. Shamirzaev, Heterostructures with diffused interfaces: Luminescent technique for ascertainment of band alignment type, J. Appl. Phys. **123**, 115701 (2018).
[23] G. M. Jacobsen, H. Bragança, Yu. I. Mazur, M. E. Ware, G. J. Salamo, B. L. Liang, G. E. Marques, V. Lopez-Richard, and M. D. Teodoro, Quantum Dots as an Active Reservoir for Longer Effective Lifetimes in GaAs Bulk, ACS Appl. Nano Mater. **7**, 15978 (2024).
[24] A. Tadjine, Y.-M. Niquet, and C. Delerue, Universal behavior of electron g-factors in semiconductor nanostructures, Phys. Rev. B **95**, 235437 (2017).
[25] A. V. Shumilin, T. S. Shamirzaev, and D. S. Smirnov, Spin Light Emitting Diode Based on Exciton Fine Structure Tuning in Quantum Dots, Phys. Rev. Lett. **132**, 076202 (2024).
[26] G. M. Jacobsen, V. A. Oliveira, B. Liang, A. Pelais, H. V. Stanchu, M. E. Ware, G. J. Salamo, Yu. I. Mazur, G. E. Marques, V. Lopez-Richard, and M. D. Teodoro, Optical spin polarization by coherent magnetoabsorption generation, Nanoscale **17**, 17174 (2025).
[27] B. Urbaszek, X. Marie, T. Amand, O. Krebs, P. Voisin, P. Maletinsky, A. Högele, and A. Imamoglu, Nuclear spin physics in quantum dots: An optical investigation, Rev. Mod. Phys. **85**, 79 (2013).
[28] P. Klenovský, P. Steindl, and D. Geffroy, Excitonic structure and pumping power dependent emission blue-shift of type-II quantum dots, Sci. Rep. **7**, 45568 (2017).
[29] M. E. Ware, E. A. Stinaff, D. Gammon, M. F. Doty, A. S. Bracker, D. Gershoni, V. L. Korenev, S. C. Bădescu, Y. Lyanda-Geller, and T. L. Reinecke, Polarized Fine Structure in the Photoluminescence Excitation Spectrum of a Negatively Charged Quantum Dot, Phys. Rev. Lett. **95**, 177403 (2005).
